# Ethyl ({5-[5′-(2-eth­oxy-2-oxoeth­oxy)-4,4′′-difluoro-1,1′:3′,1′′-terphenyl-4′-yl]-1,3,4-oxadiazol-2-yl}sulfan­yl)acetate

**DOI:** 10.1107/S1600536812005028

**Published:** 2012-02-10

**Authors:** Hoong-Kun Fun, Suhana Arshad, S. Samshuddin, B. Narayana, B. K. Sarojini

**Affiliations:** a X-ray Crystallography Unit, School of Physics, Universiti Sains Malaysia, 11800 USM, Penang, Malaysia; b Department of Studies in Chemistry, Mangalore University, Mangalagangotri 574 199, India; c Department of Chemistry, P. A. College of Engineering, Nadupadavu, Mangalore 574 153, India

## Abstract

In the title compound, C_28_H_24_F_2_N_2_O_6_S, the whole mol­ecule is disordered over two sites with refined occupancies of 0.778 (3) and 0.222 (3). The central benzene ring makes dihedral angles of 56.0 (4), 34.5 (4) and 70.9 (4)°, respectively, with the two terminal benzene rings and the 1,3,4-oxadiazole ring in the major component of the disordered mol­ecule. The corresponding angles in the minor component are 59.7 (16), 25.6 (13) and 75.5 (14)°. In the crystal, mol­ecules are linked *via* C—H⋯F, C—H⋯N, C—H⋯O and C—H⋯S hydrogen bonds into a three-dimensional network. In addition, C—H⋯π inter­actions are observed.

## Related literature

For a related structure and background to terphenyls and their oxadiazole derivatives, see: Fun, Arshad *et al.* (2011 ▶); Fun, Chia *et al.* (2011 ▶); Fun *et al.* (2012 ▶); Samshuddin *et al.* (2011 ▶). For bond-length data, see: Allen *et al.* (1987 ▶). For the stability of the temperature controller used for data collection, see: Cosier & Glazer (1986 ▶).
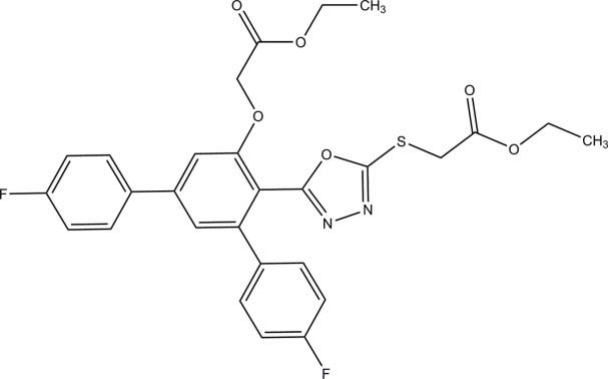


## Experimental

### 

#### Crystal data

C_28_H_24_F_2_N_2_O_6_S
*M* *_r_* = 554.55
Triclinic, 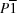

*a* = 8.2721 (8) Å
*b* = 10.274 (1) Å
*c* = 16.2342 (16) Å
α = 81.058 (2)°
β = 82.987 (2)°
γ = 83.646 (2)°
*V* = 1346.8 (2) Å^3^
*Z* = 2
Mo *K*α radiation
μ = 0.18 mm^−1^
*T* = 100 K
0.42 × 0.24 × 0.12 mm

#### Data collection

Bruker SMART APEXII DUO CCD area-detector diffractometer
Absorption correction: multi-scan (*SADABS*; Bruker, 2009 ▶) *T*_min_ = 0.928, *T*_max_ = 0.978
25187 measured reflections
7034 independent reflections
5142 reflections with *I* > 2σ(*I*)
*R*_int_ = 0.041

#### Refinement

*R*[*F* ^2^ > 2σ(*F* ^2^)] = 0.061
*wR*(*F* ^2^) = 0.196
*S* = 1.01
7034 reflections
506 parameters
99 restraints
H-atom parameters constrained
Δρ_max_ = 0.56 e Å^−3^
Δρ_min_ = −0.66 e Å^−3^

### 

Data collection: *APEX2* (Bruker, 2009 ▶); cell refinement: *SAINT* (Bruker, 2009 ▶); data reduction: *SAINT*; program(s) used to solve structure: *SHELXTL* (Sheldrick, 2008 ▶); program(s) used to refine structure: *SHELXTL*; molecular graphics: *SHELXTL*; software used to prepare material for publication: *SHELXTL* and *PLATON* (Spek, 2009 ▶).

## Supplementary Material

Crystal structure: contains datablock(s) global, I. DOI: 10.1107/S1600536812005028/is5061sup1.cif

Structure factors: contains datablock(s) I. DOI: 10.1107/S1600536812005028/is5061Isup2.hkl

Supplementary material file. DOI: 10.1107/S1600536812005028/is5061Isup3.cml

Additional supplementary materials:  crystallographic information; 3D view; checkCIF report

## Figures and Tables

**Table 1:** Hydrogen-bond geometry (Å, °) *Cg*1 is the centroid of the C7–C9/C16/C17/C22 ring.

*D*—H⋯*A*	*D*—H	H⋯*A*	*D*⋯*A*	*D*—H⋯*A*
C8—H8*A*⋯F1^i^	0.95	2.36	3.307 (8)	174
C15—H15*A*⋯N2^ii^	0.95	2.45	3.266 (5)	144
C18—H18*B*⋯O3^iii^	0.99	2.38	3.200 (11)	140
C25—H25*A*⋯F2^iv^	0.99	2.46	3.112 (5)	123
C25—H25*A*⋯O6^v^	0.99	2.49	3.158 (9)	125
C27—H27*B*⋯F2^vi^	0.99	2.46	3.217 (7)	133
C28—H28*C*⋯S1^v^	0.98	2.85	3.700 (10)	146
C5—H5*A*⋯*Cg*1^vii^	0.95	2.76	3.579 (6)	145
C18—H18*A*⋯*Cg*1^ii^	0.99	2.62	3.456 (10)	142

Symmetry codes: (i) 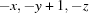; (ii) 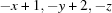; (iii) 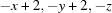; (iv) 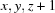; (v) 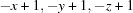; (vi) 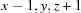; (vii) 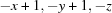.

**Fig. 1.**
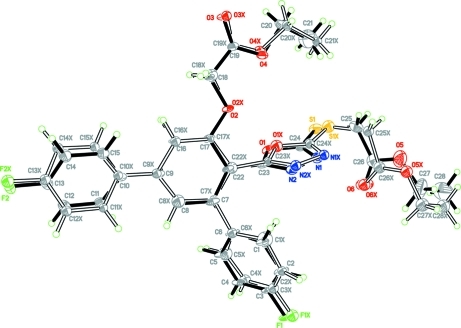
The molecular structure of the title compound, showing 30% probability displacement ellipsoids and the atom-numbering scheme. All disordered components are shown.

**Fig. 2.**
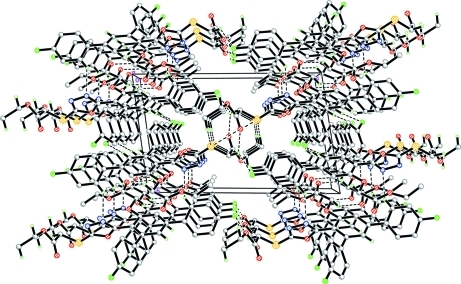
The crystal packing of the title compound. Dashed lines represent the intermolecular hydrogen bonds. Only major disordered component is shown.

## supplementary crystallographic information

### Comment

In continuation of our work on synthesis of terphenyls and their oxadiazole derivatives (Fun, Arshad *et al.*, 2011; Fun, Chia *et al.*, 2011; Fun *et al.*, 2012), the title compound is prepared and its crystal structure is reported. The starting material of the title compound was prepared from 4,4'-difluoro chalcone by several steps (Samshuddin *et al.*, 2011).

The molecular structure in shown in Fig. 1. The whole molecule of the title compound is disordered over two positions with a refined site-occupancy ratio of 0.778 (3): 0.222 (3). For the major disorder component, the central benzene ring (C7–C9/C16/C17/C22) makes dihedral angles of 56.0 (4), 34.5 (4) and 70.9 (4)° with the terminal benzene rings (C1–C6 & C10–C15) and 1,3,4-oxadiazole ring (O1/N1/N2/C23/C24), respectively. On the other hand, for the minor disorder component, the central benzene ring (C7X–C9X/C16X/C17X/C22X) makes dihedral angles of 59.7 (16), 25.6 (13) and 75.5 (14)° with the terminal benzene rings (C1X–C6X & C10X–C15X) and 1,3,4-oxadiazole ring (O1X/N1X/N2X/C23X/C24X), respectively. Bond lengths (Allen *et al.*, 1987) and angles are within normal range.

In the crystal structure (Fig. 2), the molecules are linked *via* intermolecular C8—H8A···F1, C15—H15A···N2, C18—H18B···O3, C25—H25A···F2, C25—H25A···O6, C27—H27B···F2 and C28—H28C···S1 hydrogen bonds (Table 1) into a three-dimensional network. The crystal structure are further stabilized by the intermolecular C5—H5A···*Cg*1 and C18—H18A···*Cg*1 (Table 1) interactions (*Cg*1 is the centroid of C7–C9/C16/C17/C22).

### Experimental

To a solution of 5-(4,4''-difluoro-5'-hydroxy-1,1':3',1''-terphenyl-4'-yl)-1,3,4 -oxadiazole-2(3*H*)-thione (3.82 g, 0.01 mol) in ethanol (20 ml), 10% aqueous sodium hydroxide solution (3.5 ml) was added. Ethyl chloroacetate (1 ml) was then added and heated to reflux for 4 h. The reaction mixture was then poured into ice cold water, filtered and crystallized from ethanol. The single-crystal was grown from DMF by slow evaporation method and the yield of the compound was 72%. (*M. p.*: 378 K).

### Refinement

The title compound is disordered over two positions with a refined site-occupancy ratio of 0.778 (3): 0.222 (3) and the minor disordered component were refined isotropically. All H atoms were positioned geometrically [C—H = 0.95–0.99 Å] and refined using a riding model with *U*_iso_(H) = 1.2 or 1.5 *U*_eq_(C). A rotating group model was applied to the methyl groups. The restraints of same geometries were applied for major and minor components. The same *U^ij^* parameters were also used for atom pairs C21/C28 and O1X/O6X.

### Crystal data

**Table:**

C_28_H_24_F_2_N_2_O_6_S	*Z* = 2
*M**_r_* = 554.55	*F*(000) = 576
Triclinic, *P*1	*D*_x_ = 1.367 Mg m^−^^3^
Hall symbol: -P 1	Mo *K*α radiation, λ = 0.71073 Å
*a* = 8.2721 (8) Å	Cell parameters from 9309 reflections
*b* = 10.274 (1) Å	θ = 2.5–30.0°
*c* = 16.2342 (16) Å	µ = 0.18 mm^−^^1^
α = 81.058 (2)°	*T* = 100 K
β = 82.987 (2)°	Block, pink
γ = 83.646 (2)°	0.42 × 0.24 × 0.12 mm
*V* = 1346.8 (2) Å^3^	

### Data collection

**Table:**

Bruker SMART APEXII DUO CCD area-detector diffractometer	7034 independent reflections
Radiation source: fine-focus sealed tube	5142 reflections with *I* > 2σ(*I*)
Graphite monochromator	*R*_int_ = 0.041
φ and ω scans	θ_max_ = 29.0°, θ_min_ = 1.3°
Absorption correction: multi-scan (*SADABS*; Bruker, 2009)	*h* = −11→11
*T*_min_ = 0.928, *T*_max_ = 0.978	*k* = −13→13
25187 measured reflections	*l* = −22→22

### Refinement

**Table:**

Refinement on *F*^2^	Primary atom site location: structure-invariant direct methods
Least-squares matrix: full	Secondary atom site location: difference Fourier map
*R*[*F*^2^ > 2σ(*F*^2^)] = 0.061	Hydrogen site location: inferred from neighbouring sites
*wR*(*F*^2^) = 0.196	H-atom parameters constrained
*S* = 1.01	*w* = 1/[σ^2^(*F*_o_^2^) + (0.1038*P*)^2^ + 1.0279*P*] where *P* = (*F*_o_^2^ + 2*F*_c_^2^)/3
7034 reflections	(Δ/σ)_max_ < 0.001
506 parameters	Δρ_max_ = 0.56 e Å^−^^3^
99 restraints	Δρ_min_ = −0.66 e Å^−^^3^

### Special details

**Table:**

Experimental. The crystal was placed in the cold stream of an Oxford Cryosystems Cobra open-flow nitrogen cryostat (Cosier & Glazer, 1986) operating at 100.0 (1) K.
Geometry. All e.s.d.'s (except the e.s.d. in the dihedral angle between two l.s. planes) are estimated using the full covariance matrix. The cell e.s.d.'s are taken into account individually in the estimation of e.s.d.'s in distances, angles and torsion angles; correlations between e.s.d.'s in cell parameters are only used when they are defined by crystal symmetry. An approximate (isotropic) treatment of cell e.s.d.'s is used for estimating e.s.d.'s involving l.s. planes.
Refinement. Refinement of *F*^2^ against ALL reflections. The weighted *R*-factor *wR* and goodness of fit *S* are based on *F*^2^, conventional *R*-factors *R* are based on *F*, with *F* set to zero for negative *F*^2^. The threshold expression of *F*^2^ > σ(*F*^2^) is used only for calculating *R*-factors(gt) *etc*. and is not relevant to the choice of reflections for refinement. *R*-factors based on *F*^2^ are statistically about twice as large as those based on *F*, and *R*- factors based on ALL data will be even larger.

### Fractional atomic coordinates and isotropic or equivalent isotropic displacement parameters (Å^2^)

**Table:**

	*x*	*y*	*z*	*U*_iso_*/*U*_eq_	Occ. (<1)
S1	0.58917 (13)	0.60843 (12)	0.37370 (6)	0.0401 (3)	0.778 (3)
F1	−0.1744 (5)	0.4107 (4)	0.1802 (3)	0.0420 (8)	0.778 (3)
F2	0.6675 (3)	0.8297 (3)	−0.44112 (12)	0.0638 (8)	0.778 (3)
O1	0.5304 (3)	0.6637 (3)	0.21551 (18)	0.0289 (5)	0.778 (3)
O2	0.6528 (9)	0.8792 (8)	0.0945 (4)	0.029 (2)	0.778 (3)
O3	0.9649 (11)	1.0846 (8)	0.1047 (5)	0.0337 (17)	0.778 (3)
O4	0.8094 (4)	0.9572 (3)	0.20113 (16)	0.0358 (7)	0.778 (3)
O5	0.2059 (5)	0.7253 (4)	0.51466 (19)	0.0741 (10)	0.778 (3)
O6	0.2572 (10)	0.5310 (6)	0.4679 (6)	0.0668 (17)	0.778 (3)
N1	0.3396 (4)	0.7577 (4)	0.29952 (17)	0.0334 (7)	0.778 (3)
N2	0.3012 (4)	0.7960 (4)	0.21606 (19)	0.0291 (8)	0.778 (3)
C1	0.0796 (5)	0.6815 (4)	0.1171 (5)	0.0250 (12)	0.778 (3)
H1A	0.0784	0.7725	0.1221	0.030*	0.778 (3)
C2	−0.0501 (9)	0.6090 (7)	0.1563 (6)	0.040 (2)	0.778 (3)
H2A	−0.1361	0.6473	0.1919	0.048*	0.778 (3)
C3	−0.0484 (7)	0.4809 (6)	0.1415 (5)	0.0319 (13)	0.778 (3)
C4	0.0733 (5)	0.4176 (4)	0.0933 (5)	0.0280 (9)	0.778 (3)
H4A	0.0692	0.3284	0.0853	0.034*	0.778 (3)
C5	0.2037 (5)	0.4891 (4)	0.0563 (7)	0.0252 (12)	0.778 (3)
H5A	0.2887	0.4493	0.0210	0.030*	0.778 (3)
C6	0.2101 (8)	0.6188 (7)	0.0708 (5)	0.028 (3)	0.778 (3)
C7	0.3507 (10)	0.6935 (10)	0.0303 (4)	0.027 (3)	0.778 (3)
C8	0.3921 (8)	0.7060 (7)	−0.0551 (3)	0.023 (2)	0.778 (3)
H8A	0.3313	0.6656	−0.0886	0.027*	0.778 (3)
C9	0.5218 (13)	0.7769 (12)	−0.0929 (3)	0.0246 (17)	0.778 (3)
C10	0.5623 (4)	0.7890 (4)	−0.1843 (2)	0.0263 (8)	0.778 (3)
C11	0.5427 (4)	0.6849 (3)	−0.22762 (19)	0.0378 (7)	0.778 (3)
H11A	0.5044	0.6054	−0.1975	0.045*	0.778 (3)
C12	0.5784 (5)	0.6968 (4)	−0.3139 (2)	0.0501 (9)	0.778 (3)
H12A	0.5666	0.6261	−0.3434	0.060*	0.778 (3)
C13	0.6326 (5)	0.8162 (4)	−0.35627 (19)	0.0443 (8)	0.778 (3)
C14	0.6569 (5)	0.9188 (3)	−0.31574 (18)	0.0382 (7)	0.778 (3)
H14A	0.6974	0.9976	−0.3458	0.046*	0.778 (3)
C15	0.6203 (4)	0.9036 (3)	−0.22892 (17)	0.0336 (6)	0.778 (3)
H15A	0.6355	0.9736	−0.1996	0.040*	0.778 (3)
C16	0.6144 (12)	0.8387 (11)	−0.0458 (3)	0.023 (2)	0.778 (3)
H16A	0.7008	0.8891	−0.0725	0.028*	0.778 (3)
C17	0.5768 (12)	0.8244 (12)	0.0398 (4)	0.026 (3)	0.778 (3)
C18	0.7831 (10)	0.9574 (9)	0.0570 (4)	0.0192 (17)	0.778 (3)
H18A	0.7400	1.0324	0.0171	0.023*	0.778 (3)
H18B	0.8674	0.9027	0.0254	0.023*	0.778 (3)
C19	0.8586 (8)	1.0093 (7)	0.1238 (3)	0.0250 (13)	0.778 (3)
C20	0.9103 (8)	0.9863 (6)	0.2664 (2)	0.0642 (17)	0.778 (3)
H20A	1.0281	0.9653	0.2491	0.077*	0.778 (3)
H20B	0.8916	1.0812	0.2727	0.077*	0.778 (3)
C21	0.8614 (12)	0.9047 (10)	0.3464 (3)	0.134 (3)	0.778 (3)
H21A	0.9064	0.9364	0.3920	0.202*	0.778 (3)
H21B	0.9034	0.8123	0.3437	0.202*	0.778 (3)
H21C	0.7418	0.9112	0.3569	0.202*	0.778 (3)
C22	0.4441 (7)	0.7530 (7)	0.0782 (4)	0.0209 (15)	0.778 (3)
C23	0.4165 (5)	0.7381 (5)	0.1686 (3)	0.0258 (10)	0.778 (3)
C24	0.4733 (4)	0.6828 (4)	0.2944 (2)	0.0294 (6)	0.778 (3)
C25	0.4750 (5)	0.6811 (4)	0.4553 (2)	0.0464 (8)	0.778 (3)
H25A	0.5352	0.6610	0.5055	0.056*	0.778 (3)
H25B	0.4648	0.7784	0.4389	0.056*	0.778 (3)
C26	0.3012 (7)	0.6343 (5)	0.4787 (2)	0.0499 (10)	0.778 (3)
C27	0.0381 (8)	0.6954 (8)	0.5440 (3)	0.097 (2)	0.778 (3)
H27A	−0.0017	0.6456	0.5042	0.117*	0.778 (3)
H27B	−0.0333	0.7792	0.5455	0.117*	0.778 (3)
C28	0.0273 (12)	0.6142 (10)	0.6312 (3)	0.134 (3)	0.778 (3)
H28A	−0.0872	0.5998	0.6504	0.202*	0.778 (3)
H28B	0.0704	0.6619	0.6702	0.202*	0.778 (3)
H28C	0.0917	0.5286	0.6289	0.202*	0.778 (3)
S1X	0.5488 (5)	0.6512 (4)	0.3949 (2)	0.0423 (9)*	0.222 (3)
F1X	−0.1874 (19)	0.4273 (16)	0.1937 (10)	0.038 (3)*	0.222 (3)
F2X	0.6240 (7)	0.9223 (7)	−0.4532 (3)	0.0348 (14)*	0.222 (3)
O1X	0.5170 (16)	0.6877 (13)	0.2335 (7)	0.038 (3)*	0.222 (3)
O2X	0.659 (3)	0.878 (2)	0.0940 (11)	0.013 (5)*	0.222 (3)
O3X	0.955 (4)	1.093 (3)	0.1106 (17)	0.024 (4)*	0.222 (3)
O4X	0.8537 (13)	0.9319 (12)	0.2057 (7)	0.032 (3)*	0.222 (3)
O5X	0.1312 (13)	0.7237 (9)	0.5181 (6)	0.038 (2)*	0.222 (3)
O6X	0.254 (3)	0.5344 (17)	0.4657 (16)	0.038 (3)*	0.222 (3)
N1X	0.3157 (13)	0.7979 (11)	0.3061 (6)	0.025 (3)*	0.222 (3)
N2X	0.2928 (17)	0.8299 (13)	0.2189 (7)	0.027 (3)*	0.222 (3)
C1X	0.087 (3)	0.681 (3)	0.125 (2)	0.059 (11)*	0.222 (3)
H1XA	0.1053	0.7605	0.1443	0.071*	0.222 (3)
C2X	−0.058 (3)	0.623 (2)	0.1543 (15)	0.020 (5)*	0.222 (3)
H2XA	−0.1543	0.6742	0.1728	0.024*	0.222 (3)
C3X	−0.055 (3)	0.489 (2)	0.1552 (18)	0.027 (5)*	0.222 (3)
C4X	0.069 (3)	0.422 (2)	0.1104 (15)	0.034 (6)*	0.222 (3)
H4XA	0.0684	0.3302	0.1084	0.041*	0.222 (3)
C5X	0.197 (4)	0.491 (2)	0.068 (3)	0.049 (10)*	0.222 (3)
H5XA	0.2825	0.4442	0.0366	0.058*	0.222 (3)
C6X	0.207 (3)	0.625 (2)	0.0680 (17)	0.025 (9)*	0.222 (3)
C7X	0.355 (3)	0.695 (3)	0.0340 (11)	0.020 (8)*	0.222 (3)
C8X	0.399 (4)	0.712 (4)	−0.0541 (12)	0.042 (11)*	0.222 (3)
H8XA	0.3404	0.6668	−0.0857	0.051*	0.222 (3)
C9X	0.518 (5)	0.789 (4)	−0.1000 (8)	0.022 (6)*	0.222 (3)
C10X	0.5499 (15)	0.8212 (14)	−0.1947 (7)	0.020 (3)*	0.222 (3)
C11X	0.4290 (9)	0.8101 (8)	−0.2467 (4)	0.0184 (15)*	0.222 (3)
H11B	0.3274	0.7803	−0.2213	0.022*	0.222 (3)
C12X	0.4523 (9)	0.8412 (8)	−0.3336 (5)	0.0244 (17)*	0.222 (3)
H12B	0.3701	0.8311	−0.3676	0.029*	0.222 (3)
C13X	0.5997 (10)	0.8870 (11)	−0.3683 (5)	0.0262 (19)*	0.222 (3)
C14X	0.7297 (12)	0.8863 (10)	−0.3221 (5)	0.026 (2)*	0.222 (3)
H14B	0.8349	0.9053	−0.3489	0.031*	0.222 (3)
C15X	0.7017 (11)	0.8567 (9)	−0.2352 (5)	0.0212 (16)*	0.222 (3)
H15B	0.7880	0.8607	−0.2023	0.025*	0.222 (3)
C16X	0.602 (4)	0.847 (4)	−0.0455 (11)	0.026 (8)*	0.222 (3)
H16B	0.6873	0.8998	−0.0700	0.031*	0.222 (3)
C17X	0.568 (4)	0.830 (4)	0.0420 (9)	0.010 (5)*	0.222 (3)
C18X	0.788 (5)	0.958 (4)	0.0629 (15)	0.038 (10)*	0.222 (3)
H18C	0.7416	1.0394	0.0294	0.046*	0.222 (3)
H18D	0.8664	0.9100	0.0245	0.046*	0.222 (3)
C19X	0.879 (3)	0.997 (2)	0.1281 (9)	0.018 (4)*	0.222 (3)
C20X	0.8684 (16)	1.0238 (13)	0.2688 (8)	0.028 (3)*	0.222 (3)
H20C	0.9843	1.0359	0.2723	0.033*	0.222 (3)
H20D	0.8082	1.1115	0.2537	0.033*	0.222 (3)
C21X	0.7923 (11)	0.9531 (9)	0.3500 (6)	0.0228 (17)*	0.222 (3)
H21D	0.8435	0.9750	0.3968	0.034*	0.222 (3)
H21E	0.8090	0.8573	0.3492	0.034*	0.222 (3)
H21F	0.6748	0.9808	0.3569	0.034*	0.222 (3)
C22X	0.443 (4)	0.753 (4)	0.0873 (10)	0.034 (8)*	0.222 (3)
C23X	0.4077 (19)	0.7599 (16)	0.1786 (7)	0.014 (3)*	0.222 (3)
C24X	0.4470 (15)	0.7199 (12)	0.3081 (6)	0.024 (3)*	0.222 (3)
C25X	0.4106 (13)	0.7118 (12)	0.4705 (7)	0.031 (2)*	0.222 (3)
H25C	0.4596	0.6968	0.5242	0.037*	0.222 (3)
H25D	0.3875	0.8085	0.4546	0.037*	0.222 (3)
C26X	0.2475 (15)	0.6464 (14)	0.4832 (11)	0.038 (4)*	0.222 (3)
C27X	−0.0292 (17)	0.6719 (14)	0.5411 (9)	0.046 (3)*	0.222 (3)
H27C	−0.0513	0.6190	0.4984	0.055*	0.222 (3)
H27D	−0.1160	0.7462	0.5434	0.055*	0.222 (3)
C28X	−0.0306 (17)	0.5858 (14)	0.6262 (8)	0.047 (3)*	0.222 (3)
H28D	−0.1427	0.5653	0.6466	0.071*	0.222 (3)
H28E	0.0108	0.6332	0.6660	0.071*	0.222 (3)
H28F	0.0393	0.5034	0.6210	0.071*	0.222 (3)

### Atomic displacement parameters (Å^2^)

**Table:**

	*U*^11^	*U*^22^	*U*^33^	*U*^12^	*U*^13^	*U*^23^
S1	0.0406 (5)	0.0444 (6)	0.0347 (4)	−0.0170 (4)	−0.0120 (4)	0.0121 (4)
F1	0.0311 (13)	0.0526 (19)	0.0462 (19)	−0.0355 (13)	0.0006 (11)	−0.0007 (15)
F2	0.0891 (17)	0.0798 (19)	0.0308 (10)	−0.0392 (14)	0.0001 (10)	−0.0166 (10)
O1	0.0228 (10)	0.0285 (13)	0.0334 (13)	−0.0101 (8)	−0.0010 (9)	0.0052 (11)
O2	0.027 (3)	0.026 (2)	0.036 (3)	−0.0186 (13)	0.0023 (8)	−0.0027 (8)
O3	0.028 (2)	0.032 (2)	0.044 (2)	−0.0204 (17)	0.0029 (14)	−0.0094 (15)
O4	0.0421 (17)	0.0432 (17)	0.0271 (11)	−0.0278 (15)	−0.0051 (11)	−0.0023 (10)
O5	0.064 (2)	0.112 (3)	0.0458 (16)	−0.008 (2)	−0.0025 (15)	−0.0131 (16)
O6	0.075 (3)	0.074 (3)	0.052 (2)	−0.0416 (19)	−0.0190 (14)	0.0242 (15)
N1	0.0375 (17)	0.0317 (18)	0.0322 (13)	−0.0117 (13)	−0.0028 (10)	−0.0031 (12)
N2	0.0320 (15)	0.0225 (17)	0.0340 (15)	−0.0092 (12)	−0.0042 (9)	−0.0024 (12)
C1	0.0150 (14)	0.0199 (17)	0.040 (2)	−0.0085 (9)	−0.0064 (12)	0.0024 (10)
C2	0.0196 (19)	0.044 (4)	0.056 (3)	−0.0138 (19)	−0.0043 (14)	0.001 (2)
C3	0.024 (2)	0.038 (2)	0.035 (3)	−0.0233 (15)	−0.0083 (14)	0.0075 (17)
C4	0.0262 (18)	0.0204 (18)	0.039 (3)	−0.0147 (11)	−0.0128 (15)	0.0070 (15)
C5	0.0162 (18)	0.0155 (18)	0.043 (3)	−0.0077 (9)	−0.0080 (12)	0.0054 (12)
C6	0.016 (2)	0.019 (3)	0.049 (4)	−0.0078 (11)	−0.0094 (13)	0.0092 (13)
C7	0.017 (2)	0.012 (2)	0.052 (5)	−0.0049 (9)	−0.0065 (13)	0.0023 (12)
C8	0.0141 (18)	0.0118 (18)	0.044 (3)	−0.0050 (9)	−0.0056 (10)	−0.0058 (10)
C9	0.0191 (18)	0.016 (3)	0.040 (3)	−0.0044 (17)	−0.0022 (17)	−0.0075 (18)
C10	0.0220 (14)	0.0211 (19)	0.0380 (17)	−0.0051 (13)	−0.0039 (11)	−0.0083 (14)
C11	0.0444 (16)	0.0318 (15)	0.0413 (15)	−0.0176 (12)	−0.0054 (12)	−0.0068 (12)
C12	0.072 (2)	0.0443 (19)	0.0417 (16)	−0.0276 (17)	−0.0056 (15)	−0.0150 (14)
C13	0.0555 (19)	0.050 (2)	0.0308 (14)	−0.0179 (17)	−0.0036 (13)	−0.0092 (14)
C14	0.051 (2)	0.0296 (15)	0.0348 (15)	−0.0127 (15)	−0.0040 (13)	−0.0025 (11)
C15	0.0415 (16)	0.0253 (14)	0.0361 (14)	−0.0120 (13)	−0.0018 (11)	−0.0063 (11)
C16	0.015 (2)	0.018 (2)	0.038 (3)	−0.0062 (17)	0.0027 (10)	−0.0092 (12)
C17	0.020 (3)	0.017 (3)	0.043 (3)	−0.0065 (18)	0.0002 (11)	−0.0050 (11)
C18	0.0174 (19)	0.0148 (19)	0.026 (2)	−0.0112 (10)	0.0008 (11)	−0.0011 (11)
C19	0.020 (2)	0.019 (2)	0.036 (2)	−0.0044 (18)	−0.0009 (13)	−0.0046 (11)
C20	0.103 (5)	0.071 (4)	0.0313 (17)	−0.056 (4)	−0.003 (2)	−0.015 (2)
C21	0.171 (6)	0.202 (7)	0.0420 (19)	−0.102 (5)	−0.012 (3)	0.006 (3)
C22	0.0150 (17)	0.0121 (17)	0.035 (2)	−0.0049 (8)	−0.0023 (12)	0.0024 (13)
C23	0.0229 (16)	0.018 (2)	0.0369 (19)	−0.0097 (12)	−0.0026 (12)	−0.0002 (14)
C24	0.0331 (15)	0.0274 (17)	0.0285 (15)	−0.0162 (13)	−0.0012 (11)	0.0018 (13)
C25	0.056 (2)	0.044 (2)	0.0436 (18)	−0.0117 (18)	−0.0193 (17)	−0.0051 (15)
C26	0.055 (3)	0.069 (3)	0.0245 (16)	−0.013 (2)	−0.0090 (18)	0.0073 (14)
C27	0.063 (4)	0.171 (7)	0.049 (2)	0.012 (4)	0.004 (3)	−0.010 (3)
C28	0.171 (6)	0.202 (7)	0.0420 (19)	−0.102 (5)	−0.012 (3)	0.006 (3)

### Geometric parameters (Å, º)

**Table:**

S1—C24	1.730 (3)	S1X—C25X	1.714 (10)
S1—C25	1.746 (4)	S1X—C24X	1.744 (10)
F1—C3	1.366 (4)	F1X—C3X	1.365 (14)
F2—C13	1.361 (3)	F2X—C13X	1.365 (9)
O1—C24	1.346 (5)	O1X—C24X	1.350 (12)
O1—C23	1.373 (4)	O1X—C23X	1.415 (13)
O2—C17	1.367 (5)	O2X—C17X	1.376 (12)
O2—C18	1.434 (5)	O2X—C18X	1.411 (15)
O3—C19	1.215 (5)	O3X—C19X	1.212 (15)
O4—C19	1.320 (5)	O4X—C19X	1.333 (14)
O4—C20	1.510 (5)	O4X—C20X	1.521 (13)
O5—C26	1.315 (6)	O5X—C26X	1.309 (13)
O5—C27	1.461 (7)	O5X—C27X	1.469 (13)
O6—C26	1.203 (6)	O6X—C26X	1.222 (14)
N1—C24	1.277 (5)	N1X—C24X	1.277 (12)
N1—N2	1.415 (4)	N1X—N2X	1.434 (12)
N2—C23	1.301 (5)	N2X—C23X	1.301 (13)
C1—C6	1.394 (5)	C1X—C2X	1.394 (16)
C1—C2	1.405 (5)	C1X—C6X	1.413 (15)
C1—H1A	0.9500	C1X—H1XA	0.9500
C2—C3	1.372 (7)	C2X—C3X	1.372 (15)
C2—H2A	0.9500	C2X—H2XA	0.9500
C3—C4	1.367 (6)	C3X—C4X	1.369 (15)
C4—C5	1.394 (4)	C4X—C5X	1.391 (16)
C4—H4A	0.9500	C4X—H4XA	0.9500
C5—C6	1.396 (6)	C5X—C6X	1.385 (16)
C5—H5A	0.9500	C5X—H5XA	0.9500
C6—C7	1.492 (4)	C6X—C7X	1.493 (13)
C7—C8	1.376 (5)	C7X—C8X	1.420 (14)
C7—C22	1.406 (6)	C7X—C22X	1.429 (14)
C8—C9	1.390 (5)	C8X—C9X	1.400 (15)
C8—H8A	0.9500	C8X—H8XA	0.9500
C9—C16	1.405 (5)	C9X—C16X	1.419 (15)
C9—C10	1.470 (6)	C9X—C10X	1.519 (13)
C10—C15	1.384 (5)	C10X—C15X	1.402 (12)
C10—C11	1.401 (4)	C10X—C11X	1.409 (12)
C11—C12	1.385 (4)	C11X—C12X	1.392 (9)
C11—H11A	0.9500	C11X—H11B	0.9500
C12—C13	1.401 (5)	C12X—C13X	1.380 (10)
C12—H12A	0.9500	C12X—H12B	0.9500
C13—C14	1.371 (4)	C13X—C14X	1.383 (11)
C14—C15	1.393 (4)	C14X—C15X	1.393 (10)
C14—H14A	0.9500	C14X—H14B	0.9500
C15—H15A	0.9500	C15X—H15B	0.9500
C16—C17	1.375 (5)	C16X—C17X	1.402 (13)
C16—H16A	0.9500	C16X—H16B	0.9500
C17—C22	1.415 (5)	C17X—C22X	1.434 (14)
C18—C19	1.507 (6)	C18X—C19X	1.500 (15)
C18—H18A	0.9900	C18X—H18C	0.9900
C18—H18B	0.9900	C18X—H18D	0.9900
C20—C21	1.470 (7)	C20X—C21X	1.504 (13)
C20—H20A	0.9900	C20X—H20C	0.9900
C20—H20B	0.9900	C20X—H20D	0.9900
C21—H21A	0.9800	C21X—H21D	0.9800
C21—H21B	0.9800	C21X—H21E	0.9800
C21—H21C	0.9800	C21X—H21F	0.9800
C22—C23	1.444 (6)	C22X—C23X	1.486 (14)
C25—C26	1.551 (7)	C25X—C26X	1.550 (14)
C25—H25A	0.9900	C25X—H25C	0.9900
C25—H25B	0.9900	C25X—H25D	0.9900
C27—C28	1.526 (7)	C27X—C28X	1.520 (14)
C27—H27A	0.9900	C27X—H27C	0.9900
C27—H27B	0.9900	C27X—H27D	0.9900
C28—H28A	0.9800	C28X—H28D	0.9800
C28—H28B	0.9800	C28X—H28E	0.9800
C28—H28C	0.9800	C28X—H28F	0.9800
			
C24—S1—C25	97.3 (2)	C23X—N2X—N1X	106.6 (10)
C24—O1—C23	103.0 (3)	C2X—C1X—C6X	121.8 (18)
C17—O2—C18	115.4 (5)	C2X—C1X—H1XA	119.1
C19—O4—C20	114.1 (3)	C6X—C1X—H1XA	119.1
C26—O5—C27	116.8 (5)	C3X—C2X—C1X	117.2 (18)
C24—N1—N2	105.7 (3)	C3X—C2X—H2XA	121.4
C23—N2—N1	106.4 (3)	C1X—C2X—H2XA	121.4
C6—C1—C2	119.5 (4)	F1X—C3X—C4X	120.6 (16)
C6—C1—H1A	120.3	F1X—C3X—C2X	118.2 (15)
C2—C1—H1A	120.3	C4X—C3X—C2X	120.7 (15)
C3—C2—C1	117.7 (5)	C3X—C4X—C5X	118.6 (17)
C3—C2—H2A	121.1	C3X—C4X—H4XA	120.7
C1—C2—H2A	121.1	C5X—C4X—H4XA	120.7
F1—C3—C4	118.0 (5)	C6X—C5X—C4X	123.9 (18)
F1—C3—C2	117.5 (5)	C6X—C5X—H5XA	118.0
C4—C3—C2	124.5 (4)	C4X—C5X—H5XA	118.0
C3—C4—C5	117.4 (4)	C5X—C6X—C1X	113.3 (16)
C3—C4—H4A	121.3	C5X—C6X—C7X	123.8 (19)
C5—C4—H4A	121.3	C1X—C6X—C7X	120.2 (16)
C4—C5—C6	120.5 (5)	C8X—C7X—C22X	120.4 (14)
C4—C5—H5A	119.8	C8X—C7X—C6X	118.3 (16)
C6—C5—H5A	119.8	C22X—C7X—C6X	121.1 (14)
C1—C6—C5	120.1 (4)	C9X—C8X—C7X	127.7 (17)
C1—C6—C7	120.2 (5)	C9X—C8X—H8XA	116.2
C5—C6—C7	119.5 (5)	C7X—C8X—H8XA	116.2
C8—C7—C22	118.3 (4)	C8X—C9X—C16X	110.6 (14)
C8—C7—C6	120.8 (5)	C8X—C9X—C10X	127.2 (15)
C22—C7—C6	120.9 (5)	C16X—C9X—C10X	121.9 (15)
C7—C8—C9	120.7 (4)	C15X—C10X—C11X	116.6 (8)
C7—C8—H8A	119.6	C15X—C10X—C9X	122.2 (13)
C9—C8—H8A	119.6	C11X—C10X—C9X	121.1 (13)
C8—C9—C16	121.5 (5)	C12X—C11X—C10X	122.7 (7)
C8—C9—C10	119.4 (4)	C12X—C11X—H11B	118.7
C16—C9—C10	119.1 (4)	C10X—C11X—H11B	118.7
C15—C10—C11	119.0 (3)	C13X—C12X—C11X	117.2 (7)
C15—C10—C9	120.4 (4)	C13X—C12X—H12B	121.4
C11—C10—C9	120.6 (5)	C11X—C12X—H12B	121.4
C12—C11—C10	120.7 (3)	F2X—C13X—C12X	118.5 (7)
C12—C11—H11A	119.7	F2X—C13X—C14X	118.3 (7)
C10—C11—H11A	119.7	C12X—C13X—C14X	122.8 (8)
C11—C12—C13	118.0 (3)	C13X—C14X—C15X	117.9 (8)
C11—C12—H12A	121.0	C13X—C14X—H14B	121.1
C13—C12—H12A	121.0	C15X—C14X—H14B	121.1
F2—C13—C14	118.4 (3)	C14X—C15X—C10X	122.0 (8)
F2—C13—C12	118.7 (3)	C14X—C15X—H15B	119.0
C14—C13—C12	122.9 (3)	C10X—C15X—H15B	119.0
C13—C14—C15	117.7 (3)	C17X—C16X—C9X	124.4 (16)
C13—C14—H14A	121.2	C17X—C16X—H16B	117.8
C15—C14—H14A	121.2	C9X—C16X—H16B	117.8
C10—C15—C14	121.7 (3)	O2X—C17X—C16X	123.4 (15)
C10—C15—H15A	119.2	O2X—C17X—C22X	112.6 (13)
C14—C15—H15A	119.2	C16X—C17X—C22X	123.8 (14)
C17—C16—C9	118.3 (5)	O2X—C18X—C19X	115.3 (17)
C17—C16—H16A	120.8	O2X—C18X—H18C	108.4
C9—C16—H16A	120.8	C19X—C18X—H18C	108.4
O2—C17—C16	125.8 (5)	O2X—C18X—H18D	108.4
O2—C17—C22	114.0 (5)	C19X—C18X—H18D	108.4
C16—C17—C22	120.1 (5)	H18C—C18X—H18D	107.5
O2—C18—C19	110.1 (5)	O3X—C19X—O4X	123.5 (17)
O2—C18—H18A	109.6	O3X—C19X—C18X	119.1 (17)
C19—C18—H18A	109.6	O4X—C19X—C18X	116.8 (15)
O2—C18—H18B	109.6	C21X—C20X—O4X	103.4 (10)
C19—C18—H18B	109.6	C21X—C20X—H20C	111.1
H18A—C18—H18B	108.2	O4X—C20X—H20C	111.1
O3—C19—O4	125.1 (6)	C21X—C20X—H20D	111.1
O3—C19—C18	120.5 (5)	O4X—C20X—H20D	111.1
O4—C19—C18	114.2 (4)	H20C—C20X—H20D	109.0
C21—C20—O4	108.4 (4)	C20X—C21X—H21D	109.5
C21—C20—H20A	110.0	C20X—C21X—H21E	109.5
O4—C20—H20A	110.0	H21D—C21X—H21E	109.5
C21—C20—H20B	110.0	C20X—C21X—H21F	109.5
O4—C20—H20B	110.0	H21D—C21X—H21F	109.5
H20A—C20—H20B	108.4	H21E—C21X—H21F	109.5
C7—C22—C17	120.9 (5)	C7X—C22X—C17X	113.0 (12)
C7—C22—C23	122.2 (4)	C7X—C22X—C23X	129.5 (14)
C17—C22—C23	116.8 (4)	C17X—C22X—C23X	116.9 (14)
N2—C23—O1	111.2 (4)	N2X—C23X—O1X	111.3 (10)
N2—C23—C22	129.0 (4)	N2X—C23X—C22X	130.0 (18)
O1—C23—C22	119.5 (5)	O1X—C23X—C22X	118.6 (17)
N1—C24—O1	113.8 (3)	N1X—C24X—O1X	116.0 (9)
N1—C24—S1	128.9 (3)	N1X—C24X—S1X	128.2 (9)
O1—C24—S1	117.4 (3)	O1X—C24X—S1X	115.8 (9)
C26—C25—S1	114.0 (3)	C26X—C25X—S1X	112.8 (8)
C26—C25—H25A	108.7	C26X—C25X—H25C	109.0
S1—C25—H25A	108.7	S1X—C25X—H25C	109.0
C26—C25—H25B	108.7	C26X—C25X—H25D	109.0
S1—C25—H25B	108.7	S1X—C25X—H25D	109.0
H25A—C25—H25B	107.6	H25C—C25X—H25D	107.8
O6—C26—O5	123.9 (6)	O6X—C26X—O5X	133.4 (14)
O6—C26—C25	126.9 (6)	O6X—C26X—C25X	116.7 (14)
O5—C26—C25	109.2 (4)	O5X—C26X—C25X	109.7 (10)
O5—C27—C28	111.3 (5)	O5X—C27X—C28X	109.4 (10)
O5—C27—H27A	109.4	O5X—C27X—H27C	109.8
C28—C27—H27A	109.4	C28X—C27X—H27C	109.8
O5—C27—H27B	109.4	O5X—C27X—H27D	109.8
C28—C27—H27B	109.4	C28X—C27X—H27D	109.8
H27A—C27—H27B	108.0	H27C—C27X—H27D	108.2
C25X—S1X—C24X	97.8 (6)	C27X—C28X—H28D	109.5
C24X—O1X—C23X	101.1 (9)	C27X—C28X—H28E	109.5
C17X—O2X—C18X	122.3 (15)	H28D—C28X—H28E	109.5
C19X—O4X—C20X	109.4 (12)	C27X—C28X—H28F	109.5
C26X—O5X—C27X	116.9 (10)	H28D—C28X—H28F	109.5
C24X—N1X—N2X	104.8 (9)	H28E—C28X—H28F	109.5
			
C24—N1—N2—C23	0.7 (4)	C24X—N1X—N2X—C23X	4.3 (16)
C6—C1—C2—C3	−5.1 (13)	C6X—C1X—C2X—C3X	26 (5)
C1—C2—C3—F1	179.6 (8)	C1X—C2X—C3X—F1X	171 (3)
C1—C2—C3—C4	2.1 (14)	C1X—C2X—C3X—C4X	−16 (4)
F1—C3—C4—C5	−178.0 (7)	F1X—C3X—C4X—C5X	176 (3)
C2—C3—C4—C5	−0.6 (12)	C2X—C3X—C4X—C5X	4 (5)
C3—C4—C5—C6	2.0 (12)	C3X—C4X—C5X—C6X	0 (6)
C2—C1—C6—C5	6.7 (14)	C4X—C5X—C6X—C1X	8 (6)
C2—C1—C6—C7	−178.7 (8)	C4X—C5X—C6X—C7X	170 (3)
C4—C5—C6—C1	−5.1 (14)	C2X—C1X—C6X—C5X	−22 (5)
C4—C5—C6—C7	−179.8 (8)	C2X—C1X—C6X—C7X	176 (3)
C1—C6—C7—C8	−121.1 (10)	C5X—C6X—C7X—C8X	69 (5)
C5—C6—C7—C8	53.6 (13)	C1X—C6X—C7X—C8X	−130 (4)
C1—C6—C7—C22	58.9 (13)	C5X—C6X—C7X—C22X	−116 (4)
C5—C6—C7—C22	−126.5 (11)	C1X—C6X—C7X—C22X	44 (5)
C22—C7—C8—C9	−0.7 (15)	C22X—C7X—C8X—C9X	−3 (7)
C6—C7—C8—C9	179.2 (10)	C6X—C7X—C8X—C9X	171 (4)
C7—C8—C9—C16	−0.3 (18)	C7X—C8X—C9X—C16X	3 (7)
C7—C8—C9—C10	−179.7 (9)	C7X—C8X—C9X—C10X	−172 (4)
C8—C9—C10—C15	145.6 (8)	C8X—C9X—C10X—C15X	−157 (4)
C16—C9—C10—C15	−33.8 (14)	C16X—C9X—C10X—C15X	29 (6)
C8—C9—C10—C11	−34.3 (14)	C8X—C9X—C10X—C11X	20 (6)
C16—C9—C10—C11	146.3 (10)	C16X—C9X—C10X—C11X	−154 (4)
C15—C10—C11—C12	−0.9 (5)	C15X—C10X—C11X—C12X	−4.3 (16)
C9—C10—C11—C12	179.0 (6)	C9X—C10X—C11X—C12X	179 (2)
C10—C11—C12—C13	−0.8 (6)	C10X—C11X—C12X—C13X	−1.4 (14)
C11—C12—C13—F2	−179.7 (3)	C11X—C12X—C13X—F2X	−177.9 (8)
C11—C12—C13—C14	2.4 (6)	C11X—C12X—C13X—C14X	8.8 (15)
F2—C13—C14—C15	179.9 (3)	F2X—C13X—C14X—C15X	176.8 (9)
C12—C13—C14—C15	−2.2 (6)	C12X—C13X—C14X—C15X	−9.9 (16)
C11—C10—C15—C14	1.1 (6)	C13X—C14X—C15X—C10X	3.6 (16)
C9—C10—C15—C14	−178.8 (6)	C11X—C10X—C15X—C14X	3.2 (17)
C13—C14—C15—C10	0.4 (6)	C9X—C10X—C15X—C14X	−180 (2)
C8—C9—C16—C17	2 (2)	C8X—C9X—C16X—C17X	−1 (7)
C10—C9—C16—C17	−178.8 (11)	C10X—C9X—C16X—C17X	174 (4)
C18—O2—C17—C16	−0.3 (18)	C18X—O2X—C17X—C16X	6 (6)
C18—O2—C17—C22	−177.3 (9)	C18X—O2X—C17X—C22X	−179 (4)
C9—C16—C17—O2	−179.0 (12)	C9X—C16X—C17X—O2X	174 (4)
C9—C16—C17—C22	−2.2 (19)	C9X—C16X—C17X—C22X	0 (8)
C17—O2—C18—C19	−178.8 (9)	C17X—O2X—C18X—C19X	−178 (3)
C20—O4—C19—O3	−6.6 (11)	C20X—O4X—C19X—O3X	23 (3)
C20—O4—C19—C18	167.6 (7)	C20X—O4X—C19X—C18X	−148 (3)
O2—C18—C19—O3	−175.5 (9)	O2X—C18X—C19X—O3X	−159 (3)
O2—C18—C19—O4	10.0 (11)	O2X—C18X—C19X—O4X	13 (5)
C19—O4—C20—C21	−170.4 (7)	C19X—O4X—C20X—C21X	164.7 (14)
C8—C7—C22—C17	0.3 (15)	C8X—C7X—C22X—C17X	2 (6)
C6—C7—C22—C17	−179.7 (9)	C6X—C7X—C22X—C17X	−172 (3)
C8—C7—C22—C23	−176.0 (8)	C8X—C7X—C22X—C23X	173 (3)
C6—C7—C22—C23	4.1 (13)	C6X—C7X—C22X—C23X	−1 (6)
O2—C17—C22—C7	178.4 (10)	O2X—C17X—C22X—C7X	−175 (3)
C16—C17—C22—C7	1.2 (17)	C16X—C17X—C22X—C7X	0 (6)
O2—C17—C22—C23	−5.1 (14)	O2X—C17X—C22X—C23X	13 (5)
C16—C17—C22—C23	177.7 (10)	C16X—C17X—C22X—C23X	−172 (4)
N1—N2—C23—O1	−0.2 (5)	N1X—N2X—C23X—O1X	−4.4 (18)
N1—N2—C23—C22	−173.9 (4)	N1X—N2X—C23X—C22X	179.8 (17)
C24—O1—C23—N2	−0.3 (4)	C24X—O1X—C23X—N2X	2.7 (18)
C24—O1—C23—C22	174.1 (4)	C24X—O1X—C23X—C22X	179.1 (16)
C7—C22—C23—N2	−76.8 (10)	C7X—C22X—C23X—N2X	−72 (5)
C17—C22—C23—N2	106.8 (9)	C17X—C22X—C23X—N2X	98 (3)
C7—C22—C23—O1	110.0 (8)	C7X—C22X—C23X—O1X	112 (4)
C17—C22—C23—O1	−66.4 (10)	C17X—C22X—C23X—O1X	−77 (4)
N2—N1—C24—O1	−0.9 (4)	N2X—N1X—C24X—O1X	−2.8 (16)
N2—N1—C24—S1	177.4 (3)	N2X—N1X—C24X—S1X	176.4 (10)
C23—O1—C24—N1	0.8 (4)	C23X—O1X—C24X—N1X	0.3 (18)
C23—O1—C24—S1	−177.7 (3)	C23X—O1X—C24X—S1X	−179.0 (11)
C25—S1—C24—N1	−5.1 (3)	C25X—S1X—C24X—N1X	4.1 (14)
C25—S1—C24—O1	173.2 (3)	C25X—S1X—C24X—O1X	−176.7 (11)
C24—S1—C25—C26	68.0 (3)	C24X—S1X—C25X—C26X	69.8 (11)
C27—O5—C26—O6	1.2 (8)	C27X—O5X—C26X—O6X	−1 (3)
C27—O5—C26—C25	−177.7 (3)	C27X—O5X—C26X—C25X	−175.0 (11)
S1—C25—C26—O6	25.1 (8)	S1X—C25X—C26X—O6X	27 (2)
S1—C25—C26—O5	−156.0 (3)	S1X—C25X—C26X—O5X	−158.5 (11)
C26—O5—C27—C28	84.4 (7)	C26X—O5X—C27X—C28X	83.6 (15)

### Hydrogen-bond geometry (Å, º)

*Cg*1 is the centroid of the C7–C9/C16/C17/C22 ring.

**Table:**

*D*—H···*A*	*D*—H	H···*A*	*D*···*A*	*D*—H···*A*
C8—H8*A*···F1^i^	0.95	2.36	3.307 (8)	174
C15—H15*A*···N2^ii^	0.95	2.45	3.266 (5)	144
C18—H18*B*···O3^iii^	0.99	2.38	3.200 (11)	140
C25—H25*A*···F2^iv^	0.99	2.46	3.112 (5)	123
C25—H25*A*···O6^v^	0.99	2.49	3.158 (9)	125
C27—H27*B*···F2^vi^	0.99	2.46	3.217 (7)	133
C28—H28*C*···S1^v^	0.98	2.85	3.700 (10)	146
C5—H5*A*···*Cg*1^vii^	0.95	2.76	3.579 (6)	145
C18—H18*A*···*Cg*1^ii^	0.99	2.62	3.456 (10)	142

Symmetry codes: (i) −*x*, −*y*+1, −*z*; (ii) −*x*+1, −*y*+2, −*z*; (iii) −*x*+2, −*y*+2, −*z*; (iv) *x*, *y*, *z*+1; (v) −*x*+1, −*y*+1, −*z*+1; (vi) *x*−1, *y*, *z*+1; (vii) −*x*+1, −*y*+1, −*z*.

## References

[bb1] Allen, F. H., Kennard, O., Watson, D. G., Brammer, L., Orpen, A. G. & Taylor, R. (1987). *J. Chem. Soc. Perkin Trans. 2*, pp. S1–19.

[bb2] Bruker (2009). *SADABS*, *APEX2* and *SAINT* Bruker AXS Inc., Madison, Wisconsin, USA.

[bb3] Cosier, J. & Glazer, A. M. (1986). *J. Appl. Cryst.* **19**, 105–107.

[bb4] Fun, H.-K., Arshad, S., Samshuddin, S., Narayana, B. & Sarojini, B. K. (2011). *Acta Cryst.* E**67**, o3372. 10.1107/S1600536811048471 PMC3239015 22199864

[bb5] Fun, H.-K., Chia, T. S., Samshuddin, S., Narayana, B. & Sarojini, B. K. (2011). *Acta Cryst.* E**67**, o3390. 10.1107/S1600536811048719 PMC3239031 22199879

[bb6] Fun, H.-K., Hemamalini, M., Samshuddin, S., Narayana, B. & Sarojini, B. K. (2012). *Acta Cryst.* E**68**, o163. 10.1107/S1600536811053037 PMC3254504 22259448

[bb7] Samshuddin, S., Narayana, B. & Sarojini, B. K. (2011). *Molbank*, **2011**, M745.

[bb8] Sheldrick, G. M. (2008). *Acta Cryst.* A**64**, 112–122. 10.1107/S0108767307043930 18156677

[bb9] Spek, A. L. (2009). *Acta Cryst.* D**65**, 148–155. 10.1107/S090744490804362X PMC2631630 19171970

